# Geochemical modeling and multivariate statistical evaluation of trace elements in arsenic contaminated groundwater systems of Viterbo Area, (Central Italy)

**DOI:** 10.1186/2193-1801-3-237

**Published:** 2014-05-08

**Authors:** Giuseppe Sappa, Sibel Ergul, Flavia Ferranti

**Affiliations:** Dipartimento di Ingegneria Civile, Edile ed Ambientale, Sapienza, Università di Roma, Via Eudossiana, 18, 00186 Rome, Italy

**Keywords:** Drinking water, Arsenic, Geochemical modeling, PCA, Trace elements

## Abstract

Contamination of groundwater by naturally occurring arsenic has recently become a disturbing environmental problem in Viterbo area, Central Italy. Arsenic concentrations in most of the public supply networks exceed the maximum allowable limit of 10 μg/l (WHO) for drinking water. The primary purpose of this paper is to obtain a better understanding of the factors contributing to the high levels of As in water supply networks. This study focuses on (a) the determination of basic hydrochemical characteristics of groundwater, (b) the identification of the major sources and processes controlling the As contamination in public supply networks, (c) to find out possible relationships among the As and other trace elements through principal component analysis (PCA). Groundwater samples from public water supply wells and springs were collected and analysed for physico-chemical parameters and trace elements. Springs and well water samples are predominantly of the Na–HCO_3_, Na –Ca–HCO_3_ and Ca–HCO_3_ types and the highest arsenic concentrations were observed in Na–HCO_3_ type water. Eh-pH diagrams reveal that H_2_AsO_4_^−^ and HAsO_4_^2−^, As(V) arsenate, are the dominating As species highlighting slightly to moderately oxidizing conditions. Geochemical modeling indicates that arsenic-bearing phases were undersaturated in the groundwater, however most of the samples were saturated with respect to Fe (i.e. magnetite, hematite and goethite) and Al (diaspore and boehmite) oxide and hydroxide minerals. Concentrations of As, Li, B, Co, Sr, Mo, U and Se are highly correlated (r > 0.7) with each other, however in some groundwater samples As show also good correlations (r > 0.5) with Fe and Mn elements reflecting the relationships among the trace elements result from different geochemical processes. Evaluation of the principal component (PCA) analysis and geochemical modeling suggest that the occurrence of As and other trace element concentrations in groundwater are probably derived from (i) weathering and/or dissolution of volcanic source aquifer materials and (ii) adsorption/desorption processes on the Fe and Al oxide and hydroxide minerals.

## Introduction

Groundwater resources are generally less susceptible to pollution than surface water and are considered the best supply for drinking water, however in many parts of the world people suffer from poor drinking water quality (Chapman 1992; Foster et al. 1997). The quality of groundwater depends on the composition of recharging water and the mineralogy of the geological formations in the aquifers. The impact of human activities and the environmental parameters may also affect the geochemical mobility of certain constituents in groundwater (Chenini and Khmiri 2009). In Italy, 85% of drinking water is drawn from underground sources, especially in Viterbo area, central Italy, 94% of water used in this area rely on groundwater in the aquifers. In the last decades, the attention of legislators have increased on water and groundwater sources quality, in particular, many laws have been enacted with the purpose of preventing or at least mitigate the presence of pollutants in water. Recently, in the province of Viterbo, Central Italy, the government declared a “state of emergency” due to the presence of “high levels” of arsenic in drinking water. Preliminary initiatives have been undertaken by Italian National Institute of Health (ISS) and Optimal Territorial Area Authorities n°1 (ATO) to develop technical solutions, plant and/or management to reduce the arsenic concentrations in groundwater used for domestic purposes. The presence of arsenic and other toxic trace elements in water supply networks is a threat to population and agricultural activities (Roychowdhury et al. 2002; Meharg and Rahman 2003Shakeel and Amal 2011). The annual reports of National Research Council (1999, 2001) affirmed the limit of arsenic in drinking water at 50 μg/l, however, the US federal drinking water standard, or maximum contaminant level (MCL), was brought down to 10 μg/l. According to the World Health Organization (WHO), the provisional limit of arsenic in drinking water is 10 μg/l, and the same limit was adopted by the European commission (WHO 2006). The same limit has been take place in the Italian legislation (Legislative Decree 31/2001 “Implementation of Directive 98/83/EC on the quality of water intended for human consumption”).

Investigations in the last few years have shown that As mobilization can occur in many aquifers and concentrations can exceed the drinking-water quality standards in different hydrogeological conditions. Mechanisms responsible for these high As concentrations have been reported in many studies. High concentrations of naturally occurring As, in groundwater, is associated with the presence of geothermal systems and/or volcanic-sedimentary rock aquifers (Ballantyne and Moore 1988; Webster and Nordstrom 2003), and the mobilization of As is still open to many interpretations (Casentini and Pettine 2010). Volcanic degassing, interaction with deep-rising fluids and leaching of ore deposits may also important factors influencing the natural arsenic enrichment in groundwater circulating in active volcanic areas and geothermal fluids (Piscopo et al. 2006; López et al. 2012). Arsenic occurrence can take place through a combination of natural processes (e.g., weathering reactions, biological activity, leaching process, redox conditions in the subsurface environment, different water–rock interactions) as well as anthropogenic activities including coal mining and its combustion (Charlet and Polya 2006; Smedley and Kinniburgh 2002; Bose and Sharma 2002; Nickson et al. 2000). Arsenic is predominantly released from rocks with primary or secondary As or As-bearing minerals due to physical, chemical or microbiological weathering into aqueous environments. To understand (As) enrichment in water systems, the identification of geochemical parameters are the most important tools to better understand the occurrence and genesis of high As concentrations in groundwater, which are predominantly due to release from geogenic resources. The study of major and trace elements also allows us to evaluate water quality indices which are the important parameters for public health (Anawar et al. 2003Barbieri et al. 2013). This will help to identify the origin and the processes leading the high concentrations of toxic elements, and hence to develop possible mechanisms of their removal from solution, providing at the same time new approaches on the quality of groundwater (Bratus et al. 2006; Parisi et al. 2011; Vivona et al. 2006; Kumar et al. 2010).

This paper presents an integrated study on the occurrence and the distribution of arsenic and other trace elements in public water supply networks of Viterbo area, Central Italy. The main objective of the paper is to understand the control of geochemical processes on the As enrichment in groundwater and its relationship with other trace element concentrations. In order to better understand the implications of the mentioned geochemical processes for water quality, a sampling survey was carried out on drinking water supply networks, which covers 231 different sources including 153 wells and 78 springs. A detailed investigation was carried out on the collected samples based on physico-chemical parameters (pH, temperature, electrical conductivity, etc.), major ion and trace element chemistry. Then, conventional graphical plots, principal component analysis and geochemical modelling techniques were applied to evaluate the geochemistry of As and other trace elements and the mechanisms of their release into groundwater. Results from this work will help to design regional-scale studies of ground-water quality and to find out appropriate remediation techniques minimizing elevated levels of naturally occurring contaminants.

### Geological and hydrogeological setting of the study area

**Figure 1:**
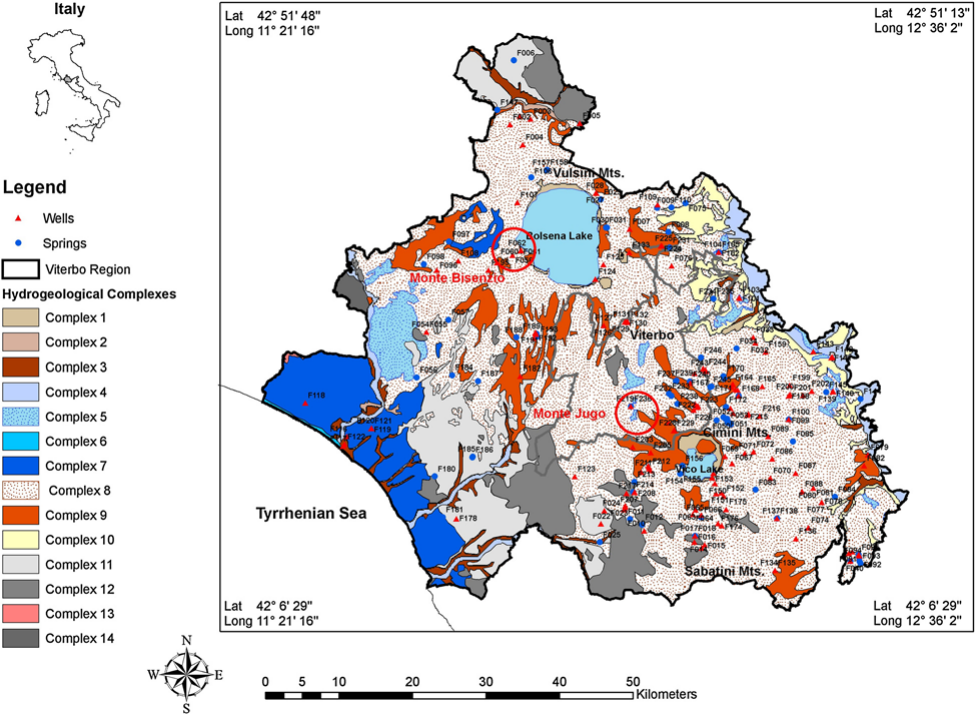
**Simplified hydrogeological map of study area and location of springs and wells: (1) Recent deposits (Oligocene); (2) Detritic complex (Pleistocene-Oligocene); (3) Alluvial complex (Pleistocene-Oligocene); (4) Alluvial deposits (Pleistocene-Oligocene); (5) Travertines (Pleistocene-Oligocene); (6) Sand dunes (Pleistocene-Oligocene); (7) Fluvial lacustrine deposits (Oligocene); (8) Pyroclastic complex (Pliocene-Pleistocene); (9) Lavas and lithoidal ignimbrites; (Pliocene-Pleistocene); (10) Heterogeneous clastic deposits (Pleistocene); (11) Pliocene Clays; (12) Clayey-marly Flysch complex with interbedded lithoids (Cretaceous-Miocene); (13) Cretaceous pelagic limestone (Cretaceous); (14) Metamorphic complexes (Palaeozoic).**

The Province of Viterbo, Central Italy, is located between the Tyrrhenian Sea coast and the Central Apennines mountains, shown in Figure 1., and was formed by two different volcanic activities in the late Pliocene - Pleistocene period: the acidic volcanic cycle of Tuscany Magmatic Province and K-alkaline volcanic cycle of the Roman Magmatic Province (Barberi et al. 1994). These volcanic formations host several aquifer systems due to the high porosity and permeability that characterize volcanic rocks. In the study area, continuous and generally unconfined volcanic and discontinuous several perched aquifers have been found. Most of the springs are related to the perched aquifers and generally discharge less than 0.01 m^3^/s. The Pliocene–Quaternary magmatic activity in the peri-Tyrrhenian sector of Italy has produced several geothermal anomalies (Della Vedova et al. 1984; Mongelli et al. 1989). The western side of Viterbo town reserves several thermal springs, which were known in Roman times and, nowadays, they are still exploited. The thermal springs of Viterbo area are related to the regional circulation of groundwater in the Mesozoic limestone aquifer (Minissale and Duchi 1988). The thickness of the volcanic aquifer (i.e. fresh waters) decreases in the thermal area where it includes more layers of travertine deposits (Piscopo et al. 2006). However, our paper considers the hydrogeochemical data of springs and shallow aquifers (temperature ranges from 10 to 25°C) currently supplying drinking water networks of Viterbo area. The presence of this important thermal groundwater circuit influences the hydrochemical properties of fresh groundwater, used for drinking supply. Three different geological districts emerge within the study area from north to south: the Vulsino district, around Bolsena Lake, located in the northernmost part, the Cimino-Vico district around Vico Lake and the Sabatino district near to lake of Bracciano (Figure 1). The outcropping rocks include mainly volcanic and volcanoclastic formations, different in age and chemical composition.

The Vulsini volcanic district is located at the northwestern end of the potassic Roman co-magmatic region, which was developed along the Tyrrhenian coast of Central Italy during Quaternary (Figure 1). The Vulsini volcanic complex include entire series of potassic rock types, with a predominance of trachytes and phonolites in terms of erupted volumes and mostly characterized by Plinian pumice-fall and ash-pumice flow deposits from larger explosive eruptions (Palladino et al. 2010). The volcanic complexes show high permeability due to the high porosity and fissures. These permeable terrains infiltrate meteoric waters and feed aquifers, located at different depths. Vulsino Basin was formed by the following hydrogeological units: lacustrine and fluvial alluvial deposits, pyroclastics, lavas, lithoid ignimbrites and volcanic-sedimentary deposits. The main shallower aquifer is located in the volcanic deposits and rests on a clay substratum consisting relatively fresh and low salinity waters. The lithotypes with low permeability, composed of sedimentary formations characterized by shallow marine to continental clays, sands, and conglomerates (Upper Miocene – Quaternary) and Upper Cretaceous – Oligocene flysch formations, which correspond to an aquiclude separating at least two main reservoirs. The Vulsino volcanic aquifer feeds the Bolsena Lake, which is the biggest volcanic lake in Europe. The deeper aquifer, confined between the impermeable formations (Ligurides and Neogene marine sediments) underlying the volcanic products and the less permeable volcanic rocks, is characterized by relatively high salinity thermal waters (Pagano et al. 2000).

The Cimino and Vico volcanic districts are different from each other, both their evolution and type of the magmas they produced. The volcanism in Cimino district is related to the acidic-felsic cycle of the Tuscany Magmatic Province consisting SiO_2_ rich magma, while Vico district shows the K-alkaline cycle volcanism of the Roman Magmatic Province (Coli et al. 1991; Perini et al. 2000). The Vico complex, located at south part of the Cimino volcanic complex, consists of a strato-volcano with a central caldera depression housing lake Vico (Figure 1). This complex was characterized by explosive volcanic activity, which was developed between 0.8 and 0.4 Ma ago. The products of Vico volcanic activity include leucitites, phono-tephrites and leucite-phonolites, while Cimino complex are mainly composed of latites and trachytes (Borghetti et al. 1983). The basement of Cimini and Vico volcanic constituted by sedimentary rocks, the Upper Cretaceous-Oligocene flysch and the Triassic- Paleogene carbonate rocks, (Cimarelli and De Rita 2006). According to previous studies, a continuous volcanic aquifer, discharges mainly into streams and springs, and several limited discontinuous perched aquifers are found in the area (Boni et al. 1986; Capelli et al. 2005; Baiocchi et al. 2007). The mean yield of the volcanic aquifer has been estimated to be between 5 and 7 m3/s. The aquifer system of Cimini and Vico volcanic area is limited by the Pliocene-Pleistocene sedimentary complex on its eastern edge and by the Upper Cretaceous-Oligocene Flysch on its western and south-western sides (Figure 1). A second deeper carbonate aquifer, located in the thick sequence of Mesozoic limestones, has been also found in the area, which consist of the Triassic-Palaeogene carbonate rocks hosting a thermal reserviour (Chiocchini et al. 2010Baiocchi et al. 2012). The volcanic aquifer rests on the carbonate aquifer and separated by low-permeability Pliocene-Pleistocene and Upper Cretaceous-Oligocene sedimentary rocks (Baiocchi et al. 2007). The thermal water of carbonate aquifer is characterized by high salinity and temperature. In the west part of Viterbo, the volcanic basement has been uplifted and reduced the thickness of sedimentary rocks. The thermal waters rise up from the carbonate aquifer through the normal faults and joints and undergo mixing with cold waters (Piscopo et al. 2006).

The Sabatini volcanic district belongs to the potassic Roman comagmatic region of quaternary age that extends along the Tyrrhenian coast (Central Italy) (Figure 1). The location of the major volcanic districts along a NW–SE tectonic trend is linked to the extensional faulting started during the Pliocene that is related to the opening of the Tyrrhenian back arc basin (De Rita et al. 1983). This area characterized by the presence of numerous cold and thermal waters and CO_2_- rich gas emissions due to the post-orogenic magmatic activity that occurred from Pliocene to Quaternary, in response to tectonic movements associated with the opening of the Tyrrhenian Sea (Minissale 2004). The main hydrogeological patterns are related to different aquifers: 1) a deeper one located in Mesozoic anhydritic-carbonate formations, and 2) shallow aquifer(s) hosted in the volcanic and sedimentary Plio- Quaternary deposits which locally may show a relatively high permeability (Dall’Aglio et al. 1994).

## Methodology

In the present research, the existing monitoring data, obtained from Italian National Institute of Health (ISS), for 231 individual domestic water supply wells and springs, was employed for geochemical modeling and statistical analysis to identify the occurrence and distribution of arsenic in the aquifers of Viterbo area. Hydrogeochemical characterization of groundwater was evaluated by means of physico-chemical analysis on the collected samples to identify the chemical characteristics and their relation with existing quality of groundwater of each municipality. The study approach includes conventional graphical plots and principal component analysis (PCA) of the hydrochemical data to define the geochemical evaluation of groundwater based on the ionic constituents, hydrochemical facies with distinct characteristics and factors controlling groundwater quality. During sampling, from 2007 to 2009, physico-chemical parameters of 231 groundwater samples (i.e. T, EC, TDS and pH) were determined in the field using PC 300 Waterproof Hand-held meter. These samples were analyzed only for major ions, Arsenic and Fluoride concentrations. The analysis were carried out at the Geochemistry Laboratory of Sapienza University of Rome and in the laboratory of National Institute of Health (ISS). Water samples were filtered through cellulose filters (0.45 μm). Each sample was divided into two subsamples: the first had stored at 4°C and been used to determine their major and minor constituents, with a Dionex DX-120 ion chromatograph (reliability ±2%). A Dionex CS-12 column was used for determining cations (Na^+^, K^+^, Mg^2+^, Ca^2+^), while a Dionex AS9-SC column was used for anions (SO_4_^−−^ HCO_3_^−^, Cl^−^, NO_3_^−^). The analytical accuracy of these methods ranged from 2% to 5%. Bicarbonate content was measured by titration with 0.1 N HCl using colour turning method with methyl orange as indicator.

**Figure 2:**
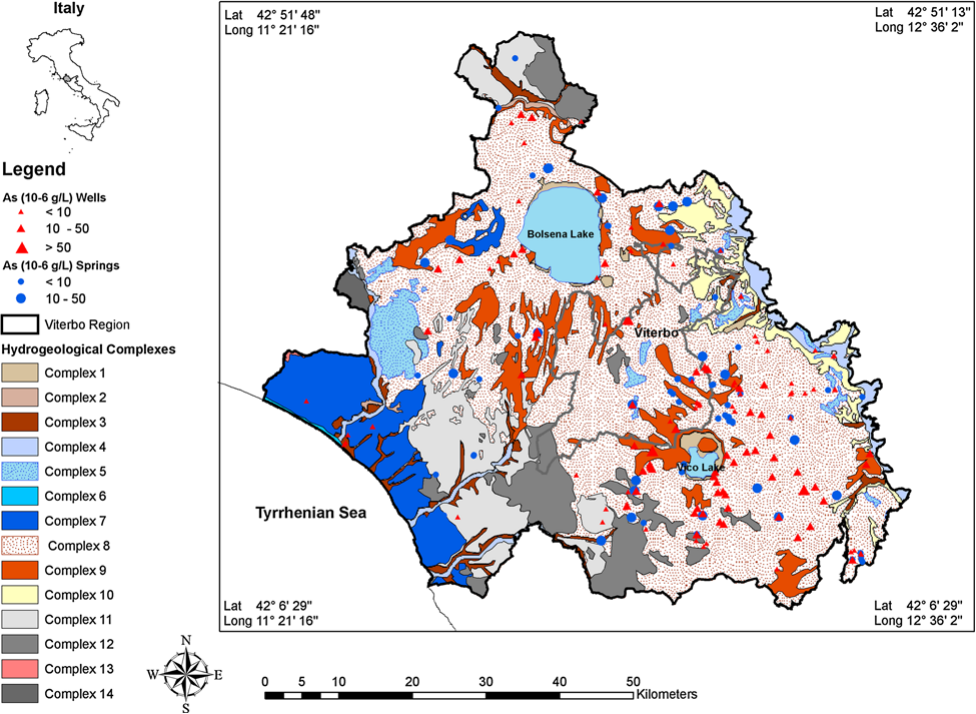
**Arsenic distribution in springs and groundwater.**

To understand As enrichment and its relationship with other trace elements, a new sampling survey was carried out in 2012. However, trace element concentrations were measured from only the seven most biggest and important drinking water supply wells in the study area. These wells are located in Monte Bisenzio area, near Vulsino district, and Monte Jugo area belonging to Cimino Vico districts (Figure 1). The concentration of trace elements were measured at different pumping rates (27) on the groundwater samples from Monte Jugo and Monte Bisenzio areas. Groundwater samples were inserted acid-washed polyetheylene bottles and acidified with concentrated nitric acid (Ultrapur, Merck, v/v) to pH < 2 and stored at 4°C and analyzed by inductively coupled plasma mass spectrometry, ICP-MS, Plasmaquad 3 Vg Elemental, (reliability ±2%) to identify trace elements. The relationship between the concentrations of arsenic were correlated with major ions and trace elements measured in groundwater to identify the source and mechanism of arsenic release in the aquifers systems of Viterbo area. All of the available arsenic data sets have been collected and incorporated into a Geographical Information System (GIS) system for the production of environmental contamination map to highlight distribution of As in monitored wells and springs (Figure 2). Garmin eTrex 20 GPS device was used for field data collection, which generally has an accuracy of ±4 m. For the identification of hydro-chemical facies Geochemistry Software AqQA) was employed. Trilinear diagram of Piper (1944) diagrams has been used to define different hydrochemical facies (Piper 1944). The chemical analysis data of the spring and well water samples have been plotted on the Piper diagram. The PHREEQC software was provided, using WATE4QF database, to compute aqueous speciation and fluid-mineral equilibrium. The estimated saturation indexes of relevant minerals (SI) are approximate due to analytical and activity concentration uncertainties; they are assumed to be ± 0.5 accurate (Parkhurst and Appello 1999). Different chemical parameters, including pH, Eh, temperature, electrical conductivity (EC), total dissolve solids (TDS), Ca, Mg, Na, K, HCO_3_, Cl, SO_4_, NO_3_, F, As and other trace elements (such as Li, V, Cr, Fe, Se, Mo, Zn, Co, Mn, Al), were used in the calculations. Principal component analysis (PCA) was applied to reduce the data sets and to identify relationships among the variables responsible for the source of groundwater contamination.

## Results and discussion

### Physicochemical parameters and major constituents

Summary statistics of physicochemical parameters (mean, median, maximum, minimum values and standard deviation values) of well and spring water samples and and guideline values of (WHO 2006) for drinking water are shown in Table 1. The pH values of spring and groundwater samples range from 6 to 8.7 indicating slightly acidic to alkaline nature and the values are in the range of WHO guideline limits (6.5 – 9.2). The mean temperature of groundwater and springs range from 15.4 to 17.1. Total dissolved solids (TDS) and electrical conductivity (EC) show a wide variation from 100.2 to 1477.5 mg/l and 134 to 1603 μS/cm in groundwater samples, and from 86 to 715.4 mg/l and 65 to 894 μS/cm in spring samples. Most of the samples show TDS values below 500 mg/l and can be considered as fresh waters, however few samples are classified as brackish water according to the WHO guidelines. The ionic dominance pattern of the water samples for cations and anions is Ca^++^ > Na^+^ > K^+^ > Mg^++^ and HCO_3_^−^ > Cl^−^ > SO_4_^−^ > NO_3_^−^. However, high NO_3_^−^ concentrations were observed in spring water samples (up to 100 mg/l) exceeding the permissible limit of 50 mg/l of WHO (2006) guideline values for drinking water. Calcium concentrations in groundwater samples vary between 10 and 221 mg/l (mean 42 mg/l Table 1), while in spring water samples range from 7 to 132 mg/l. Magnesium concentrations in both water samples are generally low (range from 3 to–40 mg/l; mean: 10.2 mg/l in groundwater and 7.4 mg/l in springs). The maximum acceptable limits for Mg is 50 mg/l and for the Ca 75 mg/l. The Na and K concentrations vary from 8 to 104 mg/l and 1 to 71 mg/l in groundwater samples and 8 to 55 mg/l and 1-39 mg/l in springs, respectively. All water samples fall within the guideline levels (<200 mg/l) for drinking water. Bicarbonate concentrations exhibit a wide range: in the groundwater samples ranging from 47 to 992 mg/l (mean: 206 mg/l; Table 1), whereas the concentrations are lower in spring water samples ( mean: 150 mg/l). The Cl concentrations in the investigated water samples are found in the range of 8-148 mg/l and the highest values were observed in groundwater samples. There are no samples in excess of the permissible limit of 250 mg/l for chloride. The SO_4_^−^ concentration in water samples range from 2 to 137 mg/l with minimum and maximum values, respectively, and all sampled waters are not exceed the permissible WHO guideline value of 250 mg/l. Major ion composition is probably controlled by water-rock reaction (i.e. dissolution of rock forming minerals), where the volcanic formations are the most dominant formations in the area investigated.

**Table 1:** **Summary statistics of physicochemical parameters (mean, median, maximum minimum and standard deviation (SD) values) of spring and well water samples**

		Wells					Springs				
WHO guideline values	Physico-chemical parameters	Min	Max	Mean	Median	SD	Min	Max	Mean	Median	SD
75-200	Ca (mg/l)	10.0	221.0	42.0	27.0	38.8	7.0	132.0	32.8	21.5	31.2
50-150	Mg (mg/l)	3.0	40.0	10.2	8.0	6.6	3.0	18.0	7.4	7.0	3.2
200	Na (mg/)	8.0	104.0	22.9	20.0	13.4	8.0	55.0	18.8	17.0	8.3
200	K (mg/l)	1.0	71.0	19.6	20.0	12.3	1.0	39.0	15.3	17.5	9.2
N.S	HCO_3_^−^ (mg/l)	47.0	992.0	206.4	163.0	138.3	46.0	416.0	150.0	122.5	90.2
250	SO_4_^2−^ (mg/l)	2.0	137.0	15.2	9.0	18.8	3.0	53.0	11.6	8.5	9.6
250	Cl (mg/l)	8.0	148.0	19.9	15.0	17.8	8.0	54.0	17.3	14.0	9.1
1.5	F^−^ (mg/l)	0.0	3.2	0.8	0.8	0.6	0.0	2.7	0.6	0.4	0.6
50	NO_3_ (mg/l)	1.0	87.0	14.6	13.0	12.2	1.0	100.0	18.4	15.0	18.6
1000	TDS (mg/l)	100.2	1477.5	351.0	287.4	220	86.0	715.4	271.9	224.3	155.7
1500	EC (μS/cm)	134	1603	434.7	372	258.8	65.0	894.0	342.9	293.0	189.2
6.5-9.2	pH	6.0	8.7	7.0	7.0	0.5	6.2	8.1	6.9	6.9	0.4
N.S	T (°C)	10.4	25.5	17.1	16.7	2.3	9.9	21.4	15.4	15.4	2.1
10 μg/l	As (μg/)	1.0	57.0	14.3	11.0	12.6	1.0	45.0	9.0	7.5	9.1
N.S	V (μg/)	1.0	76.0	15.5	12.0	12.3	2.0	51.0	13.1	10.0	10.2
-	*Eh* (V)	0.23	0.66	0.3	0.28	0.08	0.23	0.58	0.31	0.31	0.06
-	*pe*	3.9	10.1	5.4	5.4	0.95	4.0	11.4	5.3	4.9	1.3

### Trace elements

**Figure 3:**
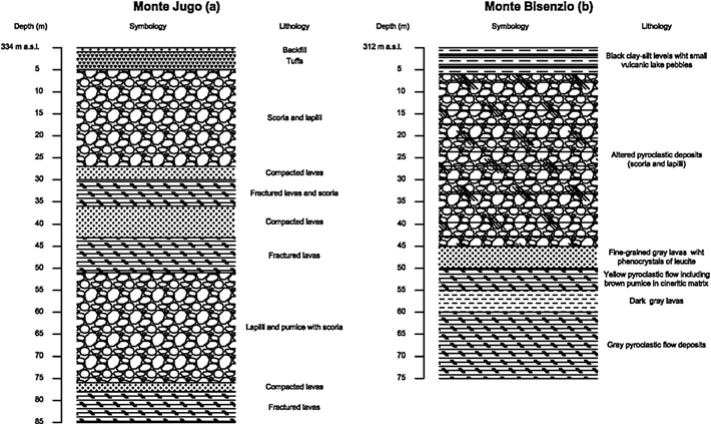
**a and b Stratigraphic units of the studied wells.**

Trace element studies were carried out by many researchers on contaminated waters in order to determine the origin of the pollution (Drever 1997; Langmiur 1996; Appelo and Postma 1993). Understanding the distribution of arsenic and other related toxic trace elements in drinking water is essential to identify contamination mechanism and to the develop suitable remediation technologies identifying high-risk areas (Parisi et al. 2011; Giammanco et al. 1998; Aiuppa et al. 2005; Buschmann et al. 2007). To evaluate the safety of water for drinking, concentrations of various trace elements, including Li, Al, B, Be, Co, Cr, F, As, Cu, Fe, Mn, Mo, Ni, Rb, U, Se, Sr, V, and Zn were determined in the most seven important water supply wells and the results were compared with the guidelines for drinking-water quality established by WHO (2006) (Table 2). A series of various trace elements, including Li, Al, B, Be, Co, Cr, F, As, Cu, Fe, Mn, Mo, Ni, Rb, U, Se, Sr, V, and Zn were determined in the studied groundwater. The concentrations of the elements are summarized in Table 2. The arsenic concentrations cover a wide range and; of the 231 water samples collected most water samples exceed the WHO (2006) guideline value of 10 μg/l for drinking water. The distribution of As concentrations in the studied water samples is shown in Figure 2. In groundwater samples, As concentrations range between 1 and 57 μg/l (mean: 14.3 μg/l), in springs between 1 and 45 μg/l (mean: 9 μg/l) (Table 1). In addition, among the determined analytes, concentrations exceeding the World Health Organization recommended drinking water limits were found for F (>1.5 μg/l) and Fe (>300 μg/l). Nickel values varies between 0.01 and 5.6 μg/l, which is below the drinking water guideline value (20 μg/l). The maximum concentrations of Cu and Zn were 91.5 and 71.5 μg/l, respectively. These values are also below the maximum permissible limit of WHO (2006) guideline values. Cobalt concentrations were also relatively low ranging from 0.28 to 1.82 μg/l. From the groundwater samples investigated, two samples showed elevated value of boron (>800 μg/l), and these values exceed the WHO (2006) drinking water guideline values of 500 μg/l. Vanadium concentration in groundwater ranges from 27.9 to 44.96 mg/l and the highest values were observed in Monte Bisenzio groundwater samples. Uranium concentrations varied between 2.7 and 13.8 μg/l not exceeding the WHO (2006) guideline permissible limits. The Fe concentration varies between 2.4 and 814.5. In general, Fe concentration was low in the studied groundwater samples and the highest concentration was observed in groundwater sample from Monte Bisenzio well exceeding the WHO (2006) guideline value (300 μg/l). Groundwater samples show also high values of Li (9-66.8 μg/l), Sr (199.5-1228.6 μg/l), Rb (30.9-169.6 μg/l) and Mn (0.16-110.5 μg/l), while the rest of trace element concentrations are generally low including Be (0.35-1.19 μg/l), Al (4.62-18.04 μg/l), Mo (0.7-2.65 μg/l), Cr (0.07-1.39 μg/l) and Se (2.7-4.60 μg/l) (Table 2). The presence of As and other trace elements such as V, Mo, U, B, F, Sr are probably related to the circulation of groundwater in the volcanic formations, which is found in most of the study area. Volcanic rocks, especially ashes, are often implicated in the generation of high-As waters. The occurrence of these elements are presumed to have the same origin derived from volcanic source aquifer materials (i.e. volcanic glass, altered tuffs and sediments). Elevated arsenic concentrations in drinking water supplies in several locations within the United States, Argentina, Greece, Turkey, Chileand Italy have been associated with volcanic rocks and ash-flow tuffs (Casentini et al. 2010; Johannesson and Tang 2009; Welch et al. 2000). The volcanic materials, especially tuffs that have undergone different types of alteration including mainly secondary silica, iron/manganese oxides, aluminum hydroxides, and various clay mineral phases. The new mineral phase plays an important role for the mobilization of As and other trace elements in terms of different mechanisms such as dissolution of volcanic glass and adsorption/desorption processes on the secondary mineral phases. Figures 3a and 3b shows the stratigraphic units of the studied wells in Monte Jugo and Monte Bisenzio areas, respectively. The stratigraphy of the wells are peresnted by well depths. As can be seen from the mentioned figures, the wells mainly composed of tuffs and pyroclastic materials. We conclude that the groundwater flowing through an aquifer composed dominantly of volcanic rocks and the weathered products generated from them are characterized by elevated concentrations of these elements.

**Table 2:** **Statistical summary (mean values) of selected trace elements of groundwater**

Monitored wells								
Trace elements (μg/l)	MJ1	MJ2	MB1	MB2	MB3	MB4	MB5	WHO limits (μg/l)
Li	9.8	9.0	66.9	16.2	30.4	66.1	33.3	N.S
Be	0.35	0.45	1.19	0.35	0.44	1.12	0.77	N.S
B	93.0	79.6	832.0	145.1	319.9	840.7	381.4	500
Al	5.4	11.9	18.0	4.6	6.4	7.0	10.3	200
V	33.8	38.5	32.5	40.0	34.3	27.9	44.8	N.S
Cr	0.61	1.4	0.07	0.37	0.20	0.10	0.07	50
Mn	0.16	0.35	110.50	30.71	11.49	99.86	99.11	400
Fe	2.4	11.3	114.1	321.8	4.6	36.8	814.5	300
Co	0.29	0.28	1.84	0.38	0.93	1.82	0.81	N.S
Ni	0.00	5.96	0.92	0.01	0.00	1.91	0.06	20
Cu	0.43	91.55	2.47	0.00	0.00	0.46	0.74	2000
Zn	0.01	16.64	2.76	13.05	0.00	1.03	71.50	3000
As	16.73	14.35	13.09	14.50	7.70	14.11	15.12	10
Se	4.60	3.32	3.49	4.00	2.90	4.19	2.70	10
Rb	30.9	34. 6	169.6	73.7	124.2	165.6	101.3	N.S
Sr	324.9	273.4	1228.77	199.5	586.4	1082.7	532.9	N.S
Mo	2.34	2.17	1.35	1.30	0.70	1.10	2.65	70
U	6.26	2.71	12.87	3.38	7.63	10.65	6.76	15

### Hydrochemical facies & distribution of arsenic

**Figure 4:**
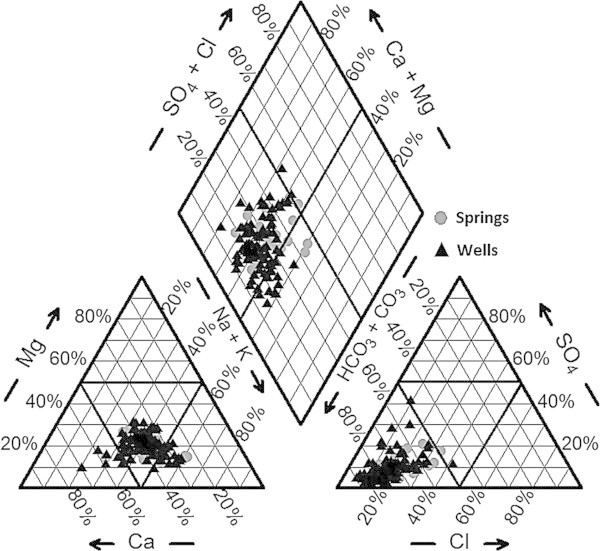
**Piper diagram of sampled waters.**

**Figure 5:**
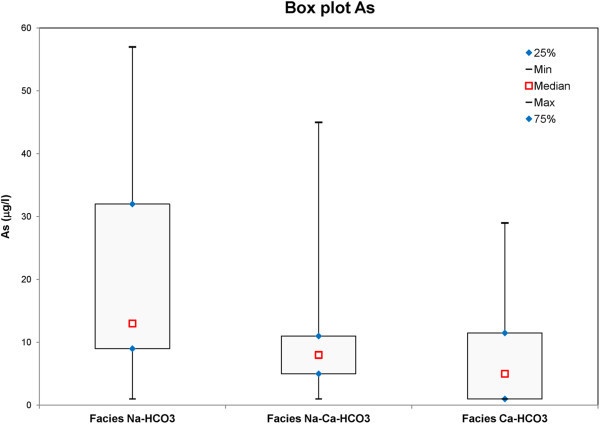
**Box plot of mean, median, maximum and minimum values of arsenic distributions versus hydrochemical facies.**

The classification of groundwater was studied by plotting the concentrations of major cations and anions in the Piper trilinear diagram to identify hydrogeochemical processes controlling groundwater chemistry (Figure 4). Major ion concentrations in meq/l for each spring and well water samples are reported as percentages of the total anion and cation content (Piper 1944). The trilinear plots suggest that among anions HCO_3_^−^ has a clear dominance. Among cationic species Ca and Na dominate in the groundwater samples. In Figure 4, the plot shows that most of the samples fall within the central part of the cation triangle reflecting the absence of any dominance among the alkali (Na + K) and alkaline earth (Ca + Mg) cations, which indicates the mixing of Ca–HCO_3_ and Na-HCO_3_ facies. According to Piper diagram, the distribution of hydrochemical facies of groundwater that occur in the Viterbo area can be classified into three groups: a) Facies A-Na- HCO_3_ type water, b) Facies B- No dominant type, Na- Ca-HCO_3_ (water type in which none of the ions is dominant and/or results of mixing of two or more different facies) and Facies C- Ca – HCO_3_ type. The occurrence of these water types in the aquifers may be due to the interactions between groundwater and different rocks with mineralogical compositions along the groundwater flow paths. From the plots, it is clearly seen that some samples appear in the cation triangle show a tendency towards the alkaline earth composition, however, the water samples which are closer to the Na + K vertex are related to the volcanic aquifers highlighting enrichment in K derived from the alkaline-potassic rocks. The higher concentration of Ca^2+^ Na^+^ and HCO^3−^ in the groundwater may be due to the dissolution of plagioclase feldspars in the rocks resulting in the release of these elements responsible for the various hydro-chemical facies. From the whole samples, 40% of the groundwater from Viterbo province show a composition of Na-HCO_3_ type. Besides, 20% of the samples shows Na-Ca-HCO_3_ hydro facies, while 40% samples have a composition of Ca-HCO_3_. Statistic parameters describing the arsenic distribution for each group of hydrochemical facies of water samples are shown in Figure 5. Evaluation of mean, median, maximum and minimum levels of arsenic depicted in the box plots, indicate the following relationships between hydrochemical facies and arsenic concentrations: (i) Na- HCO_3_ water type (Facies A), from wells and springs, are characterized by elevated As concentrations (mean value: As > 10 μg/l), exceeding the permissible limit for drinking waters; (ii) The Na-Ca-HCO_3_ water type, labelled as Facies B, from wells are characterized by elevated arsenic concentrations above the limit, while the samples from springs show low arsenic contents (mean value: As < 10 μg/l); (iii) Ca-HCO_3_ water type, Facies C, highlights low arsenic concentrations below the permissible limit (mean value: As <10 μg/l).

### Arsenic speciation and geochemical modeling

**Figure 6:**
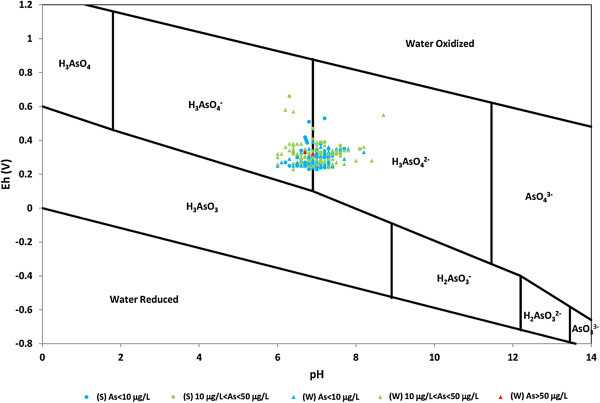
**Eh-pH diagram of the arsenic species in the system As–O–H.**

The two forms of arsenic, arsenate and arsenite are commonly found in ground water (Masscheleyn et al. 1991). Under oxidising conditions, H_2_AsO_4_^−^ is dominant at low pH, while at higher pH, HAsO_4_^2_^ and AsO_4_^3−^ becomes dominant. Under reducing conditions, pH up to 9.2, As(III) species H_3_AsO_3_ is predominant, while H_2_AsO_3_^−^ is predominant from pH 9.2 to 12 (Welch and Stollenwerk 2003). The Eh-pH diagram of arsenic species shows that arsenate As(V) is the dominant As species, as H_2_AsO_4_^−^ and HAsO_4_^2−^, in groundwater samples (Figure 6). Similarly, PHREEQC speciation modeling revealed that As(V) species in the groundwater samples are predominantly H_2_AsO_4_^−^ and HAsO_4_^2−^. Redox potential (Eh) and pH are the most important factors controlling As speciation. Many of the redox processes occur at mineral surfaces and are associated with adsorption/desorption processes. A number of studies have demonstrated that arsenic species are strongly sorbed by oxide and hydroxide minerals, especially iron, aluminum, and manganese oxyhydroxides. The adsorption depends on pH and other solution properties (Arai et al. 2001; Stollenwerk 2003). Positive levels of Eh (> +2 mV) were measured in most of the groundwater samples highlighting the oxidizing conditions. (Table 1). The measured Eh values were converted to *pe* to identify the possible redox sensitive species present in the studied water samples using the following expression:1

where F is the Faraday constant (F = 96.490 KJ ve - eq) and T is the temperature in K. The pe values of water samples ranges from 3.9 to 11.4 with min and max values, respectively, however most of the samples fall in the range of 5 to 7 indicating the iron and manganese reduction (Table 1).

Mineralogy (i.e. water-rock interaction) can control the adsorption, desorption and transport of arsenic and other trace elements, and plays an important role in the mechanism of environmental contamination. Thus, geochemical modeling technique was applied to model groundwater evolution by dissolution and precipitation of mineral phases and its relation to the mobilization of As in groundwater. Calculated saturation indexes for selected minerals are presented in Table 3, which may suggest different mineral–solution interactions, precipitation–dissolution and adsorption–desorption processes. All sampled waters are strongly undersaturated with respect arsenolite, claudetite and fluorite oxides and siderite minerals indicating that As should generally remain dissolved after mobilization. Besides, groundwater samples are also undersaturated with respect to carbonate (i.e calcite and dolomite) and sulphate (i.e. gypsum and anhydrite) minerals. Undersaturation with respect to carbonate and arsenic bearing minerals suggest that the groundwater has short residence time and natural equilibrium with these minerals is not reached. On the contrary, most of the groundwater is supersaturated with respect to Al hydroxides such as diaspore, boehmite and gibbsite (Table 3). Groundwater samples show also supersaturation with respect to ferric oxides (hematite, magnetite, maghemite, magnesioferrite, hercynite) and hydroxides (goethite, lepidocrocite, ferrihydrite), suggesting that both Al and Fe mineral phases are probably potential As adsorbents. The results show that Fe-oxides and -oxyhydroxides can precipitate, providing sites for adsorption, according to the following reaction:

**Table 3:** **Saturation index values of various mineral phases**

Samples		MJ1	MJ2	MB1	MB2	MB3	MB4	MB5
**Saturation Index Values of Minerals**	Arsenolite (As_2_O_3_)	−47.95	−55.92	−57.39	−50.39	−51.62	−55.43	−49.84
	Claudetite (As_2_O_3_)	−47.66	−55.64	−57.11	−50.11	−51.34	−55.14	−49.56
	Boehmite (γ-AlOOH)	−1.45	0.61	0.56	−0.71	−1.10	0.14	−1.76
	Diaspore (α-AlOOH)	0.31	2.40	2.34	1.07	0.68	1.90	0.02
	Goethite (FeOOH)	1.96	3.86	5.96	4.07	3.85	5.24	2.45
	Lepidocrocite (γ-FeOOH)	1.31	3.32	5.39	3.51	3.29	4.60	1.90
	Magnetite (Fe_3_O_4_)	6.53	11.68	17.86	12.70	11.94	15.87	7.95
	Gibbsite α-Al(OH)_3_	−1.08	1.02	0.96	−0.31	−0.70	0.52	−1.35
	Hematite (Fe_2_O_3_)	6.28	10.08	14.28	10.50	10.06	12.84	7.27
	Siderite (FeCO_3_)	−4.64	−3.27	−1.55	−2.43	−3.04	−1.49	−3.93
	Magnesioferrite Mg(Fe^3+^)_2_O_4_	−3.13	2.33	6.61	1.60	1.23	4.95	−2.26
	Maghemite (γ-Fe_2_O_3_)	2.99	−1.03	7.13	3.37	2.93	5.55	0.16
	Fluorite (CaF_2_)	−0.89	−1.86	−1.87	−0.15	−0.03	−0.38	−0.04
	Ferrihydrite (∼FeOOH)	−0.79	1.09	3.19	1.30	1.09	2.48	−0.31
	Hercynite (Fe^2+^Al_2_O_4_)	−3.56	1.91	3.78	−0.13	−1.23	2.40	−3.74

The adsorption of arsenic is strong at acidic to neutral conditions, however, increases in pH will result in desorption of arsenic from oxide surfaces and a resultant increase in dissolved concentrations (Fuller et al. 1993). These processes are considered to have been responsible for the release of arsenic in oxidizing aquifers, whereas under reducing condition the reductive dissolution takes place (Dzombak and Morel 1990). As a result of the pH dependence of arsenic adsorption, changes in ground-water pH can promote adsorption or desorption of arsenic. According to calculated saturation indexes it was considered two different mechanism that can lead to the release of arsenic in groundwater (a) the first is the development of high pH, and hence mineral weathering, leading the desorption of adsorbed arsenic (i.e. arsenate species) from natural mineral oxides, or prevents arsenic from being adsorbed in the first place (b) the second is the direct dissolution of mineral phases, derived from volcanic materials, presented in the aquifer system.

### Principal component analyses (PCA)

Principal component analysis (PCA) is a kind of factor analysis, which is useful to reduce the number of variables in a data set to a few components or factors, that represent most of the variation in the original data simplifying multiple variable interpretation (Hair et al. 1998). These methods have been widely used to identify geochemical controls on the groundwater composition (Seyhan et al. 1985; Join et al. 1997; Hernandez et al. 1991). PCA may result from the correlation of sets of variables representing the same geological origin and/or geochemical source. The PCA was based on the eigenanalysis of the correlation matrix, and hence the Varimax rotation was adopted to maximize the variation explained by the components (Meglin 1991; Reyment and Jvreskog 1996; Everitt et al. 2011). All PCs are uncorrelated (i.e. orthogonal) to one another. Eigenvalues describe the amount of variance explained by each PC, and thus decrease with each successive PC extracted. The number of significant principal components is selected on the basis of the Kaiser criterion and only factors with eigenvalues greater than or equal to 1 were considered (Kaiser 1960). Eigenvectors (or PC loadings) indicate the relative contribution that each element makes to that PC score (Webster 2001). In the present study, two data sets of the selected physico-chemical parameters and trace elements were used for PCA to identify the main hydrogeochemical processes governing the groundwater chemistry and to find the effects of some chemical components on arsenic contamination. The first data set for PCA includes EC, TDS, pH, T, Eh, As and major ions (Ca, Mg, Na, K, HCO3, Cl, SO4, NO3, V and F) of the 231 water samples from wells and springs measured in 2007 to 2009. A second PCA was applied with the objective to identify the relationship among the trace elements and their origin. Principal component analysis (PCA) was performed using XLSTAT. The correlation between the arsenic and other trace element concentrations of groundwater was obtained in the form of Pearson correlation coefficients to find out relationships between variables and the participation of individual chemical parameters in several influence factors.

**Figure 7:**
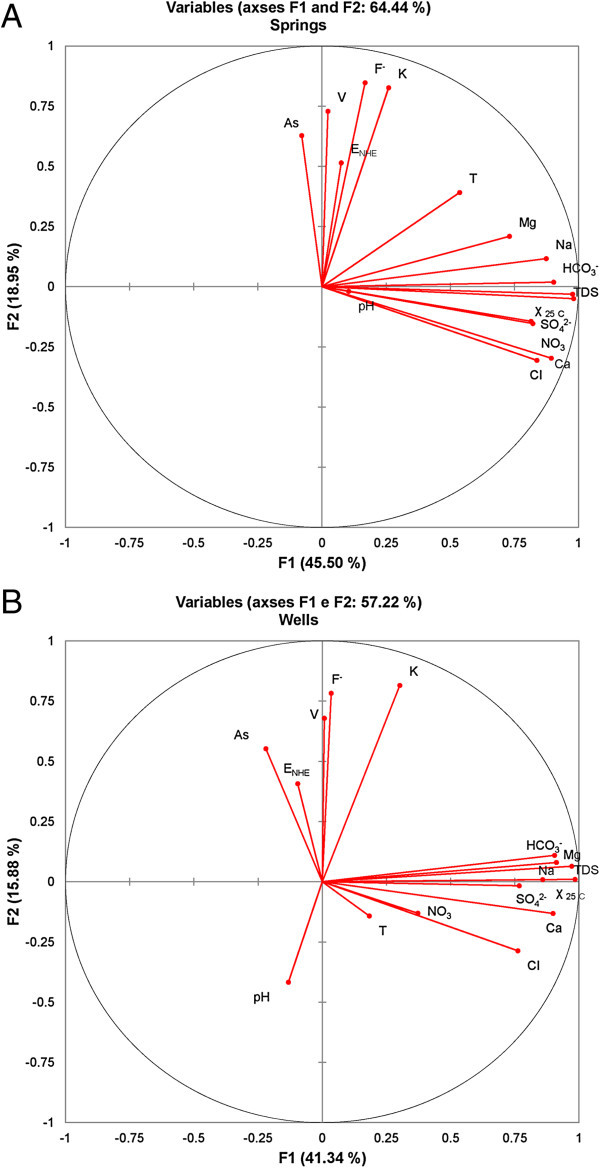
**Scatter plot between PC1 and PC2 for physico-chemical parameters of (A) springs and (B) wells.**

Table 4 summarizes the first PCA results on the data matrices of spring and groundwater samples including the loadings of each PC, percentage of variance and cumulative percentage of variance of each factor and their respective eigenvalues. The results revealed that the contribution of the first five principal components accounts for approximately 79.4% of the total variance in groundwater data. The first component (PC1), explaining 41.3% of the total variance, exhibits negative loading on As concentration, strong positive loadings on EC, Na, Mg, Ca, Cl, HCO_3_, SO_4_ and TDS, moderate loadings on Temperature and weak loadings on pH, Eh, K, F, NO_3_ and V. PC2 explains for 15.8% of the variance and represented by K, F, As and V. PC3 accounts for 8.1% of the variance and show moderately positive loading only on NO_3_ suggesting the source of pollution is possibly related to agricultural activities. PC4 and PC5 are responsible for 7.5% and 6.5% of the total variance and show moderate positive loadings for As, T, Ph and Eh, respectively. Four principal components were extracted on the spring water data matrix explaining 79.5% of the total variance. The first two PCs explain 45.4% and 18.9% of the variance, respectively, and account for the majority of the variance in the original dataset. The first one is mainly participated by EC, Na, Mg, Ca, Cl, NO_3_, HCO_3_, SO_4_ and TDS, while the second is characterized by Eh, F, As and V. PC3, which accounts for 8.5% of the variance, shows moderate positive loadings for As and pH. The fourth component (PC4) show weak positive loadings for all components. Figure 7A and B shows the loading plot for the first two PCs (PC1 and PC2) identifying different groups in springs and groundwater, respectively. Evaluation of the PC loadings (for springs and groundwater data matrices) show that most of the physico-chemical parameters with greatest positive PC1 loadings typically occurred in groundwater that has flowed through volcanic materials. Pearson correlation matrix of springs and groundwater samples show that the correlation coefficients between arsenic and physico-chemical components are very low (r < 0.5), and are statistically insignificant.

**Table 4:** **Principal component loadings of physico-chemical parameters for springs and wells including variance % and cumulative % and their respective eigenvalues**

	Springs				Wells				
	PC1	PC2	PC3	PC4	PC1	PC2	PC3	PC4	PC5
T	0.199	0.225	−0.088	0.305	0.071	−0.089	0.320	0.518	−0.405
pH	0.039	−0.011	0.699	−0.279	−0.051	−0.262	0.126	0.281	0.617
χ 25°C	0.364	−0.028	−0.006	−0.115	0.383	0.006	−0.062	−0.010	0.045
ENHE	0.028	0.296	0.152	−0.271	−0.037	0.255	0.055	0.168	0.600
Na	0.324	0.067	0.041	0.249	0.334	0.005	0.262	0.098	−0.033
K	0.096	0.475	−0.144	−0.009	0.117	0.511	−0.219	0.069	0.029
Mg	0.271	0.120	−0.249	−0.392	0.355	0.050	0.000	0.042	−0.079
Ca	0.331	−0.171	0.020	−0.188	0.349	−0.083	−0.177	−0.053	0.065
Cl	0.311	−0.176	−0.024	0.046	0.296	−0.180	0.366	−0.022	−0.044
NO3	0.305	−0.088	0.118	0.405	0.145	−0.082	0.590	−0.334	0.163
HCO3-	0.335	0.011	−0.053	−0.353	0.352	0.069	−0.253	0.025	0.040
F-	0.062	0.487	0.111	0.120	0.013	0.491	0.294	0.001	0.120
SO42-	0.302	−0.082	0.180	0.397	0.298	−0.011	−0.124	0.136	0.083
As	−0.029	0.361	0.490	0.032	−0.085	0.346	0.152	0.537	−0.121
V	0.009	0.419	−0.306	0.036	0.003	0.426	0.194	−0.432	−0.114
TDS	0.362	−0.018	−0.014	−0.161	0.378	0.040	−0.134	0.009	0.047
**Eigenvalues**	7.27	3.03	1.37	1.03	6.61	2.54	1.30	1.20	1.04
**Variance %**	45.4	18.9	8.5	6.4	41.3	15.8	8.1	7.5	6.5
**Cumulative %**	45.4	64.4	73.0	79.5	41.3	57.2	65.3	72.9	79.4

**Figure 8:**
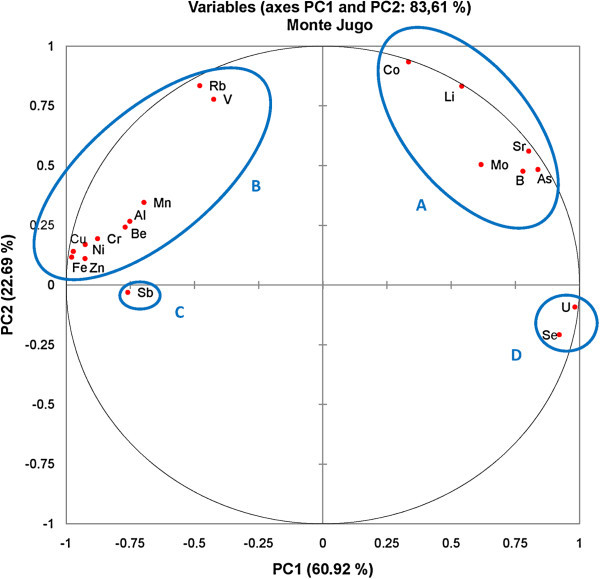
**A two dimensional sample score plot for the groundwater samples from Monte Jugo area (total PC variance 83.61%).**

**Figure 9:**
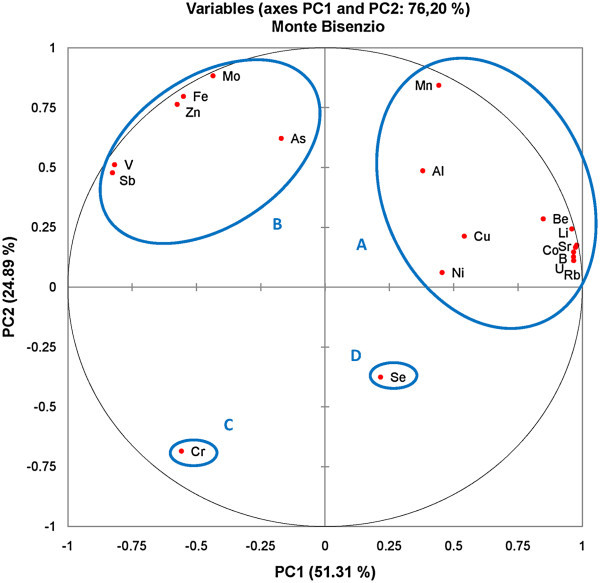
**A two dimensional sample score plot for the groundwater samples from Monte Bisenzio area (total PC variance 76.20 %).**

Principle Component Analysis (PCA) were applied for trace element data of groundwater samples from Monte Jugo and Monte Bisenzio areas to identify similarities and dissimilarities in hydrogeochemical properties and to make predictions about the As mobilization. For the groundwater samples from Monte Jugo area, the application of principal components analysis generated 7 orthogonal principal components and explained 100% of the total variation. The principal component analysis of standardized parameters resulted in seven components accounting for 60.9%, 22.6%, 9.56%, 4.1%, 1.18%, 0.85% and 0.67% of the total variance, respectively. The variables that participated in PCA as well as their obtained loadings and eigenvalues are shown in Table 5. The first component show moderately to strong positive loadings for Li, B, Co, As, Se, Sr, Mo, and U, while the second component has significant positive loadings for Li, V, Co, Rb, and moderate loadings for Sr and Mo. This indicate that these components accounted for the maximum variance of the PCA and was representative of arsenic and these trace elements release due to groundwater circulation in the volcanic materials. The third component (PC3) show weak to moderate positive loadings for Be, V, Co, Ni, As Al and Mn suggesting that the As mobilization is probably controlled by adsorption/desorption processes on the oxide/hydroxide minerals. Figure 8 shows the score plot for the first two PCs (PC1 and PC2), explaining 83.61% of the total variance within all the measured parameters. The loading plot between PC1 and PC2 has four distinct groups associated with (A) Co, Li, Mo, B, As and Sr, (B) Rb, V, Mn, Al, Be, Ni, Cu, Zn, Cr, and Fe, (C) Sb elements, and (D) U and Se (Figure 6). Table 6 presents the correlation matrix of the 19 trace element variables. Only those, with correlation values higher than 0.5, were considered. Arsenic is strongly correlated with (Pearson r > 0.50) the following trace elements: Sr (r = 0.91), Li (r = 0.84), B (r = 0.89), Co (r = 0.73), U (r = 0.77). Mo (r = 0.71), and Se (r = 0.6). High correlation coefficients between As and the mentioned trace elements show that these trace elements in groundwater had similar hydrochemical characteristics in the study area. It is very well known that volcanic rocks and sediments derived from them are also associated with elevated arsenic levels in ground water. The high concentrations of molybdenum, uranium, lithium, and boron are predominantly associated with volcanic materials and/or interaction of groundwater with geothermal waters (Aiuppa et al. 2003; Vivona et al. 2007; Arnórsson and Óskarsson 2007). The results suggest that volcanic materials are a significant source of As and these trace elements in groundwater. Groundwater, relatively more oxidizing, was characterized by greater concentrations of trace elements such as Mo, Se, B, As, and U etc. These trace elements having greatest positive PC1 loadings exhibited more soluble in oxidizing environments. According to, PHREEQC speciation modeling Mo predominant as MoO_4_^2^, selenium mostly occurred as HSeO_3_^−^ and SeO_3_^2−^ species and boron was composed of B(OH)_3_ and/or B(OH)_4_^−^ species. Uranium exists in U(IV) or U(VI) oxidation state in groundwater depending on the environmental conditions. The dominant dissolved species in the oxidized groundwater were uranyl carbonate complexes (UO_2_CO_2_^2−^; UO_2_CO_3_^4−^). In fact, the concentrations of these elements in groundwater may be due to the high dissolved oxygen content in groundwater. Table 7 summarized the PCA results of groundwater samples from Monte Bisenzio area including the loadings of each PC and their respective eigenvalues. The application of principal components analysis, generated 18 orthogonal principal components and the first seven explained 98,03% of the total variation. The first PC is responsible for 51.3% of the total variance and show strong positive loadings for Li, B, Be, Co, Rb, Sr and U. PC2 show positive loadings for all parameters except for Cr and Se and is best represented by Mn, Fe, Zn, As and V. The first two PCs (PC1 and PC2) explained 76.2% of the total variance within all the measured parameters. The loading plot between PC1 and PC2 includes four distinct groups. The identified groups were associated with the following elements: (A) Mn, Al, Cu, Ni, Be, Li, Sr, Co, Rb and U, (B) As, Mo, Fe, Zn, V and Sb, (C) Cr and (D) Se elements (Figure 9). According to Pearson correlation matrix, arsenic show positive correlations with Mn (r = 0.53), Fe (r = 0.56), and Mo (r = 0.58) whereas, correlates negatively with Cr, Co, Ni, Cu, Se, Rb, Sr, and U (Table 8). The positive correlation with Fe and Mn suggests common geogenic origin of these elements providing the presence of Fe/Mn oxyhydroxides could lead to desorption of arsenic. On the contrary, strong correlation coefficients among the other trace elements (i.e. Li, Sr, Rb, U, B, Co, Be and Co) and their close relation indicated by PCA and bivariate plots suggest the origin of these elements probably related to the volcanic source aquifer materials. The third component (PC3), explaining 7.7 % of the variance, exhibits moderate positive loadings on Ni and Se, while PC4 ( accounts for 5.4 of the variance) show moderately positive loading only for As.

**Table 5:** **Variable loadings to the first seven PCs for the groundwater samples from Monte Jugo area**

Variable	PC1	PC2	PC3	PC4	PC5	PC6	PC7
**Li**	0.541	0.833	−0.012	0.039	−0.013	0.079	−0.077
**Be**	−0.771	0.242	−0.083	0.555	−0.045	−0.176	0.002
**B**	0.779	0.476	0.201	0.320	0.095	0.085	0.082
**Al**	−0.752	0.267	0.588	−0.105	−0.029	−0.067	0.027
**V**	−0.425	0.778	0.146	−0.408	−0.124	−0.094	0.053
**Cr**	−0.878	0.194	−0.362	−0.243	0.003	−0.046	0.010
**Mn**	−0.697	0.346	0.603	0.042	−0.085	0.144	0.026
**Fe**	−0.979	0.117	−0.148	−0.019	−0.029	0.002	0.073
**Co**	0.334	0.934	0.017	−0.029	0.063	−0.082	−0.060
**Ni**	−0.926	0.170	0.039	−0.006	0.322	0.090	0.020
**Cu**	−0.972	0.141	−0.135	−0.037	0.101	0.066	−0.028
**Zn**	−0.927	0.111	−0.323	−0.098	0.114	0.036	0.029
**As**	0.838	0.484	0.104	−0.106	−0.013	0.080	0.188
**Se**	0.921	−0.208	−0.122	−0.253	−0.012	0.087	−0.145
**Rb**	−0.480	0.835	−0.161	0.116	−0.052	0.085	−0.154
**Sr**	0.802	0.561	−0.181	0.088	−0.031	−0.003	−0.024
**Mo**	0.617	0.504	−0.583	−0.073	0.065	−0.095	0.084
**Sb**	−0.760	−0.031	−0.565	0.127	−0.221	0.176	0.075
**U**	0.982	−0.092	−0.132	0.028	0.032	0.025	0.087
**Eigenvalues**	11.575	4.311	1.817	0.780	0.224	0.163	0.129
**Variance %**	60.920	22.691	9.565	4.107	1.181	0.856	0.679
**Cumulative %**	60.9	83.6	93.1	97.2	98.4	99.3	100

**Table 6:** **Pearson’s correlation matrix for trace elements measured in groundwater samples from Monte Jugo area**

	Li	Be	B	Al	V	Cr	Mn	Fe	Co	Ni	Cu	Zn	As	Se	Rb	Sr	Mo	Sb	U
**Li**	**1**	−0.206	**0.827**	−0.203	0.390	−0.323	−0.085	−0.437	**0.955**	−0.359	−0.403	−0.410	**0.843**	0.335	0.461	**0.909**	**0.743**	−0.415	0.452
**Be**		**1**	−0.343	0.550	0.300	0.627	0.573	0.785	−0.037	**0.718**	**0.758**	0.702	−0.609	**−0.906**	0.637	−0.416	−0.330	0.675	**−0.758**
**B**			**1**	−0.381	−0.078	**−0.745**	−0.238	**−0.740**	0.694	−0.595	**−0.717**	**−0.750**	**0.891**	0.508	0.018	**0.879**	0.585	−0.680	**0.716**
**Al**				**1**	0.667	0.528	**0.961**	0.685	0.013	**0.750**	0.685	0.542	−0.429	**−0.803**	0.468	−0.569	−0.658	0.214	**−0.844**
**V**					**1**	0.575	0.634	0.501	0.595	0.487	0.498	0.457	0.082	−0.483	**0.773**	0.035	0.080	0.180	−0.522
**Cr**						**1**	0.444	**0.940**	−0.108	**0.830**	**0.936**	**0.974**	−0.655	**−0.749**	0.608	−0.552	−0.209	**0.827**	**−0.839**
**Mn**							**1**	0.637	0.081	**0.714**	0.643	0.482	−0.341	**−0.789**	0.543	−0.469	−0.627	0.230	**−0.792**
**Fe**								**1**	−0.226	**0.913**	**0.984**	**0.969**	**−0.763**	**−0.913**	0.579	−0.695	−0.453	**0.834**	**−0.947**
**Co**									**1**	−0.138	−0.192	−0.206	**0.718**	0.119	0.613	**0.786**	0.676	−0.329	0.233
**Ni**										**1**	**0.957**	**0.905**	−0.682	**−0.891**	0.567	−0.666	−0.493	0.622	**−0.916**
**Cu**											**1**	**0.977**	**−0.758**	**−0.891**	0.606	−0.682	−0.449	**0.794**	**−0.948**
**Zn**												**1**	**−0.739**	**−0.815**	0.570	−0.636	−0.314	**0.854**	**−0.873**
**As**													**1**	0.665	−0.049	**0.911**	**0.715**	−0.693	**0.779**
**Se**														**1**	−0.595	0.626	0.531	−0.650	**0.922**
**Rb**															**1**	0.128	0.186	0.459	−0.537
**Sr**																**1**	**0.873**	−0.509	**0.759**
**Mo**																	**1**	−0.189	0.640
**Sb**																		**1**	−0.662
**U**																			**1**

Values in bold are different from 0 with a significance level alpha = 0.05.

**Table 7:** **Variable loadings to the first seven PCs for the groundwater samples from Monte Bisenzio area**

Variable	PC1	PC2	PC3	PC4	PC5	PC6	PC7
**Li**	0.959	0.243	0.112	0.034	−0.014	0.013	−0.011
**Be**	0.848	0.285	−0.093	0.306	−0.163	−0.074	−0.089
**B**	0.966	0.147	−0.006	0.148	−0.071	−0.016	−0.103
**Al**	0.380	0.486	−0.511	0.052	0.335	0.402	0.279
**V**	−0.818	0.512	0.034	−0.150	0.029	−0.053	0.125
**Cr**	−0.559	−0.685	−0.036	0.291	0.305	0.113	−0.112
**Mn**	0.442	0.843	0.175	0.103	0.125	0.001	−0.067
**Fe**	−0.551	0.797	0.101	−0.053	−0.029	−0.024	0.002
**Co**	0.974	0.166	0.110	−0.032	−0.068	0.014	0.020
**Ni**	0.455	0.061	0.617	−0.380	0.299	0.356	−0.202
**Cu**	0.541	0.213	−0.441	−0.093	0.535	−0.382	−0.138
**Zn**	−0.575	0.764	0.101	−0.167	−0.061	−0.044	0.064
**As**	−0.171	0.622	0.137	0.717	0.005	0.143	−0.068
**Se**	0.216	−0.375	0.709	0.254	0.297	−0.266	0.290
**Rb**	0.966	0.111	0.094	−0.125	−0.104	0.002	0.109
**Sr**	0.979	0.175	0.026	−0.063	−0.024	−0.040	0.023
**Mo**	−0.436	0.883	0.080	−0.035	0.029	−0.093	−0.008
**Sb**	−0.827	0.478	0.090	−0.029	0.080	−0.105	−0.109
**U**	0.966	0.127	−0.071	−0.125	−0.039	−0.087	0.040
**Eigenvalues**	9.7	4.7	1.4	1.0	0.74	0.57	0.30
**Variance %**	51.3	24.8	7.7	5.4	3.9	3.0	1.6
**Cumulative %**	51.3	76.2	83.9	89.4	93.3	96.4	98.0

**Table 8:** **Pearson’s correlation matrix for trace elements measured in groundwater samples from Monte Bisenzio area**

	Li	Be	B	Al	V	Cr	Mn	Fe	Co	Ni	Cu	Zn	As	Se	Rb	Sr	Mo	Sb	U
**Li**	**1**	**0.877**	**0.967**	0.423	**−0.672**	**−0.695**	**0.662**	−0.327	0.990	**0.504**	**0.507**	−0.356	0.026	0.191	**0.961**	**0.980**	−0.194	**−0.674**	**0.934**
**Be**		**1**	**0.932**	0.428	**−0.593**	**−0.617**	**0.604**	−0.268	0.850	0.185	**0.475**	−0.316	0.213	0.043	**0.799**	**0.862**	−0.135	**−0.553**	**0.843**
**B**			**1**	0.393	**−0.754**	**−0.605**	**0.559**	−0.430	0.961	0.382	**0.522**	**−0.463**	0.032	0.142	**0.924**	**0.963**	−0.291	**−0.719**	**0.936**
**Al**				**1**	−0.063	−0.394	**0.513**	0.097	0.379	0.056	**0.513**	0.077	0.240	−0.374	0.359	0.423	0.192	−0.164	0.424
**V**					**1**	0.051	0.041	**0.877**	**−0.706**	−0.291	−0.319	**0.878**	0.343	−0.323	**−0.701**	**−0.690**	**0.813**	**0.939**	**−0.684**
**Cr**						**1**	**−0.745**	−0.267	**−0.692**	−0.279	−0.331	−0.284	−0.117	0.215	**−0.703**	**−0.699**	−0.367	0.146	**−0.689**
**Mn**							**1**	0.442	**0.582**	0.360	0.404	0.380	**0.536**	−0.056	**0.501**	**0.569**	**0.578**	0.043	**0.483**
**Fe**								**1**	−0.390	−0.137	−0.168	**0.921**	**0.566**	−0.365	−0.418	−0.392	**0.947**	**0.815**	−0.428
**Co**									**1**	**0.509**	**0.472**	−0.414	−0.068	0.197	**0.985**	**0.990**	−0.270	**−0.724**	**0.956**
**Ni**										**1**	0.075	−0.137	−0.157	0.354	**0.499**	**0.470**	−0.110	−0.263	0.407
**Cu**											**1**	−0.203	−0.118	−0.082	0.451	**0.559**	−0.034	−0.293	**0.598**
**Zn**												**1**	0.448	−0.365	−0.432	−0.417	**0.946**	**0.850**	−0.449
**As**													**1**	−0.050	−0.170	−0.105	**0.585**	0.422	−0.194
**Se**														**1**	0.199	0.157	−0.346	−0.276	0.103
**Rb**															**1**	**0.981**	−0.321	**−0.759**	**0.965**
**Sr**																**1**	−0.264	**−0.714**	**0.985**
**Mo**																	**1**	**0.801**	−0.307
**Sb**																		**1**	**−0.722**
**U**																			**1**

Values in bold are different from 0 with a significance level alpha = 0.05.

## Conclusions

This paper provides information on the occurrence and the distribution of arsenic and other trace elements in the most important water supply networks of Viterbo area, Central Italy. To find out the major factors and geochemical processes affecting major and trace element concentrations in groundwater a combined geochemical modeling and principal component analysis techniques were applied. Based on the dominance of major anions and cations of water samples three hydrochemical facies identified: (i) Facies A- Na-HCO_3_ type water, (ii) Facies B- no dominant type, Na-Ca-HCO_3_ and (iii) Facies C- Ca-HCO_3_. According to speciation modeling, the dominant As species in the waters is arsenate As (V) in the forms of HAsO_4_^2−^ and H_2_AsO_4_^−^ reflecting oxidizing conditions. Geochemical modelling show that udersaturation with respect to Arsenic-bearing phases in the groundwater doesn’t contribute the arsenic mobilization. However, most of the groundwater samples show supersaturation with respect to Fe and Al oxide and hydroxide mineral phases suggesting that As concentrations in groundwater probably was controlled by adsorption/desorption processes on these minerals. Principal component analysis reveals the similarities in the concentrations of trace elements in the water samples resulting from different geochemical processes. PCA results show high positive loadings for arsenic and other trace elements such as Li, B, Rb, Co, Mo, U and Sr suggesting the primary sources of these elements are probably derived from weathering and/or dissolution of volcanic materials in oxidizing conditions. Besides, positive loadings are also observed for As, Fe and Al confirming the results of geochemical modeling that the adsorption/desorption process as a possible mechanism of As release in groundwater. The high correlation coefficients between the mentioned elements and their close relation indicated by the PCA seem to be consistent with the hypothesis that the past volcanic activity and related volcanic materials may have been a significant source of these elements.

The results show that the knowledge on geochemistry of major and trace elements and the mechanisms of their release into groundwater is important to the development effective strategies for appropriate remediation techniques minimizing elevated levels of naturally occurring contaminants. However, according to preliminary results, further investigations should be carried out concerning (i) a more detailed study on the geochemistry of aquifer rocks and sediments to confirm the volcanic materials as the source of As and other trace elements and (ii) studies of varies isotopes to develop a hydrogeochemical model and to systematize the hydrologic cycles.

## Authors’ information

GS is currently an associate professor in Engineering Geology and Applied Hydrogeology at the Department of Civil and Environmental Engineering (DICEA), Sapienza, University of Rome. SE is a research assistant in Engineering Geology at the department of Civil and Environmental Engineering, Sapienza, University of Rome. FF is a PhD student at the Department of Civil and Environmental Engineering, Sapienza, University of Rome.
